# Sheffield's Opportunity

**Published:** 1908-06-20

**Authors:** 


### HOSPITAL ADMINISTRATION.

### CONSTRUCTION AND ECONOMICS.

### SHEFFIELD'S OPPORTUNITY.

THE POSITION OF ITS VOLUNTARY HOSPITALS.

An Amazing State of Things.

We have heard a good deal in recent years of the extraordinary astuteness of the man who comes from Sheffield. If we may fairly judge that man as he is at home, from the accounts which have appeared recently of the actual condition at the present time of some of the voluntary hospitals in Sheffield, we can only say that, however astute he may be, he must lack some at any rate of the better qualities of the race. But let the reader judge for himself. The *Sheffield Telegraph* in a special article written by a member of its staff who inspected the Royal Infirmary the other day gives facts which prove that hygienically, economically and administratively this institution, if it really is as the reporter describes it, must be condemned. He begins by pointing out quite fairly and properly that the building is out of date and that to attempt its alteration would involve sheer waste of money. He shows that so little is it fitted for a hospital that, with the exception of the octagonal out-patient department, where it is relatively easy to classify the patients, he has not a good word to say for the buildings as they exist to-day. Starting with the hygienic condition he declares that from every point of view, in these days of advanced science, the sanitary arrangements are not sanitary but primitive and makeshift. There is only one operating theatre and no modern accommodation for anæsthetising patients, who lie unconscious outside the operation theatre waiting for those undergoing operation to be removed. The reporter saw children having an anæsthetic administered under conditions which should not be permitted in any well-organised hospital. The plan appears to be to place several patients who have to undergo operations in ante-rooms immediately contiguous to the theatre, where those awaiting operation are within hearing of those undergoing or being prepared for it. Such a state of things is amazing to hear of in the present day. Three centuries ago it could be witnessed in the Hôtel-Dieu, Paris, and the fact has caused this hospital and its then condition to go down to history as a byword for all time.

Why is this so?

The comforts of the patients seem to have been almost forgotten, if we may judge from the fact that there are no conveniences whatever for conveying the food from the kitchen, which is described as "a room where it is difficult to be clean and impossible to be dainty." Economy is a feature which seems to have been forgotten also, seeing that the wards are heated not from one central furnace, but by "fourteen systems of hot-water pipes." Again, the wards are described as cramped and overcrowded like the kitchen, "little, poky, badly-lighted rooms opening one into another, so that it is impossible to get a living patient into the end one or a dead patient out of it without passing through three or four wards." Is it fair to handicap a man or woman, who may otherwise be physically strong enough to face their malady under proper treatment, by admitting them to such a place as is here described? However strong constitutionally the patient may be, it is possible to make the condition of his sick chamber such that recovery may be retarded until permanent mischief is done, or death ensues. Hence, in the present day, it is absolutely unjustifiable for any responsible management to permit patients to be treated in unhygienic wards like those just mentioned. Add to bad atmospherical conditions, food prepared in a kitchen like that described, and it may well be asked, where are the medical men of Sheffield, and where is Sheffield public opinion?

A Unique Opportunity.

The *Sheffield Telegraph*, which is one of the most enterprising of the provincial papers, has published a statement which tends to show that both the Royal Infirmary and the Royal Hospital, and possibly we imagine the Jessop Hospital and the Children's Hospital, which constitute the chief voluntary medical institutions of Sheffield, need reconstruction or rebuilding or reorganisation, in the modern sense. If this be so, having regard to the murky atmosphere of the city itself, owing to the numerous and important works and factories there, it would seem to us that Sheffield has a unique opportunity of converting the present unsatisfactory state of its hospital accommodation into the most perfect system of any city in the United Kingdom. It can do it further at a cost which should prove to be infinitely smaller than that entailed by the rebuilding of many of the larger hospitals in this country in recent years. Why should not Sheffield determine to move the whole of its hospitals outside the city to a site hygienically perfect, and to retain in the city a few receiving or hospital stations where accident cases can receive first aid, and the more severe cases be kept in suitable wards for a few days? Sheffield should have its Hospital City, so designed as to afford the maximum of advantage in the way of hygienic surroundings, up-to-date construction, economical arrangement, and the best of good air. By such a plan the number of days' residence in a hospital in severe cases amongst the artisan class would be reduced to a minimum. The saving represented by the working days which would thus accrue each year to the workmen in Sheffield would represent an immense gain to each family, and collectively to the community at large.

Confidence in Sheffield!

We are glad that the Sheffield papers are unanimous in stating that the present condition of some of its voluntary hospitals demands that no makeshift schemes or tinkering shall be attempted, but that rebuilding is the proper step to take. Other urban communities all over the country in the last fifteen years have had to face the problem of hospital reconstruction and it has been wonderful to note how generously the public have come forward and how large has been the aggregate subscription, amounting as it does to many millions sterling. We are confident that the men of Sheffield will be equally generous with their money for this necessary and excellent object. The burden of the hospitals of Sheffield upon the population is relatively small at present. There are four voluntary hospitals, the aggregate expenditure of which amounts to some £36,000 per annum, or, say, to a sum equal to an annual contribution of 2s. per head per annum of the population, excluding the pauper community. Not a great sum, truly, but one which seems to have alarmed at least one of the citizens who suggests that the need of an additional yearly income of £12,000 a year for the local hospitals justifies the municipalisation of the whole of them. We have often given good reasons why the voluntary principle of hospital support is one of the most sacred trusts which have been handed to us by our forbears and sires. We have not space to pursue the question to-day, but we are glad to note that another Sheffield citizen answers the timid municipal advocate and points out the danger of the course he suggests. It may be well however to state in this connection that everywhere throughout the country the need for hospital accommodation, for those who desire to pay in whole or in part the cost of their mainteance in hospital, is manifest.

Will the Mayor Move?

Why should not the Mayor of Sheffield call a City Meeting, get a committee of representative citizens appointed, and refer to this committee the following questions:—

1. Is it desirable and possible to organise a Hospital City on some salubrious site outside the city boundary where all hospital patients can be removed for treatment; what is the estimated cost of such a plan, and the advantages and disadvantages which attach to it?

2. The desirability of establishing pay wards in connection with the voluntary hospitals for the reception of patients who can pay in whole or in part for the cost of their treatment when sick.

3. If any and what objections attach to the cost of the erection of the hospitals on the site selected for the Hospital City being advanced by the municipality to an extent equal to the difference between the sum realised by the sale of the existing sites and buildings of the voluntary hospitals, and the actual sum required to erect the new hospital buildings?

4. To prepare estimates of the combined income of the existing hospitals and of the estimated expenditure of the new establishments when erected in the Hospital City, with suggestions as to what steps should be taken to provide any increased revenues which may be found necessary for the adequate maintenance and upkeep of the new establishments.

We may say, for our own part, that we believe these suggestions are well worthy of the closest attention, and that we hope the people of Sheffield may be moved to memorialise the Mayor to appoint such a committee as we have suggested. We believe that there is no objection whatever to the municipality providing the money to erect pay wings for the reception of pay patients on a plan which would yield three per cent. interest on the outlay. This interest to be provided for in the patient's payments and to include say a half per cent. sinking fund for the redemption of the capital sunk in the buildings. In any case the atmospheric conditions of Sheffield itself, the present position and condition of its hospital buildings, and the whole of the circumstances attaching to the hospital provision at Sheffield at the moment, as described in the Sheffield papers, should be promptly faced. They present a unique opportunity to Sheffield to show the whole country how to meet modern conditions and modern scientific requirements, so far as city hospitals are concerned, by the organisation of *A Hospital City* on the lines described in The Hospital of December 2, 1905.

